# COVID-19 infection increases the risk of venous thromboembolism during pregnancy and the postpartum period

**DOI:** 10.1007/s10654-025-01268-z

**Published:** 2025-07-11

**Authors:** Anne K. Örtqvist, Jonas Söderling, Maria C. Magnus, Stine Kjaer Urhoj, Siri E. Håberg, Olof Stephansson

**Affiliations:** 1 Clinical Epidemiology Division, Department of Medicine, Karolinska Institutet, Solna, T2:02, Stockholm, SE-171 76 Sweden; 2 Department of Obstetrics and Gynecology, Visby County Hospital, Gotland, Sweden; 3 Centre for Fertility and Health, Norwegian Institute of Public Health, Oslo, Norway; 4 Department of Public Health, University of Copenhagen, Copenhagen, Denmark; 5 Statistics Denmark, Copenhagen, Denmark; 6 Department of Global Public Health and Primary Care, University of Bergen, Bergen, Norway; 7 Department of Women’s Health, Division of Obstetrics, Karolinska University Hospital, Stockholm, Sweden

**Keywords:** Venous thromboembolism, Pregnancy, COVID-19, Epidemiology, Population registers

## Abstract

Pregnant and puerperal women are at increased risk of venous thromboembolism (VTE) owing to hemostatic changes in preparation for childbirth. The objective of this study was to investigate if COVID-19 infection was associated with VTE in pregnancy or 12 weeks postpartum when considering (prophylactic or therapeutic) anticoagulant use. This population-based register study included all women in Sweden and Norway giving birth after 22 gestational weeks, with conception dates from March 2020 to 2022. A PCR-verified COVID-19 test was used as the exposure, and a VTE diagnosis during pregnancy or 12 weeks postpartum was the outcome. Non-infected women consisted of those testing negative and untested individuals. Cox regression analyses, with COVID-19 infection as a time-varying exposure, and adjusted for maternal characteristics and anticoagulant use, provided overall hazard ratios. To evaluate whether there was a particular increased risk of VTE shortly after testing positive for COVID-19, we estimated time-specific risk of VTE in the first 2, 4, 8, 12, and 16 weeks following COVID-19 infection. Data from each country were first analyzed separately and then meta-analyzed. Among 323,868 participants, 46,048 (14.2%) had COVID-19 during pregnancy, and 80 (0.2%) were diagnosed with VTE. Pregnant women with COVID-19 had a higher VTE incidence rate compared to non-infected (4.9 vs. 2.9 per 1000 person-years; adjusted overall hazard ratio [aHR] 1.26, 95% Confidence Interval [CI] 0.80-2.00). The highest risk was within two weeks of infection (aHR 4.63, 95% CI 2.71–7.90) but remained elevated up to 12 weeks post-infection (aHR 1.86, 95% CI 1.17–2.94). In the postpartum period, 8,515 (2.6%) had COVID-19, and 6 (0.07%) were diagnosed with VTE (aHR 5.17, 95% CI 2.50-10.69). Although VTE post-COVID-19 infection was rare, the infection was associated with increased VTE risk during pregnancy and postpartum, even after adjusting for anticoagulant use. These findings should contribute to the individual risk assessment when evaluating the need for prophylactic anticoagulants in pregnancy and postpartum.

**Supplementary Information:**

The online version contains supplementary material available at 10.1007/s10654-025-01268-z.

## Introduction

Early in the Coronavirus disease-19 (COVID-19) pandemic, caused by the severe acute respiratory syndrome coronavirus 2 (SARS-CoV-2), it was evident that venous thromboembolism (VTE) was a prominent feature of severe COVID-19 [1, 2]. This has been suggested to be attributed to SARS-CoV-2-related pulmonary endothelial cell dysfunction, and increased production of cytokines that trigger systemic activation of coagulation as a result of a thromboinflammatory process [3, 4]. Of note, pregnancy is a prothrombotic state owing to hemostatic alterations in preparation for childbirth [5], where immobilization, hereditary thrombophilia, or any serious infection, will increase the risk of VTE further [6].

VTE is diagnosed in approximately 0.5–2.0 per 1000 pregnant and puerperal women in Scandinavia, where about half occur during the first 12 weeks post-partum [7, 8]. Prophylactic anticoagulation (thromboprophylaxis) with low molecular weight heparin (LMWH) is recommended throughout pregnancy and postpartum in women with pre-specified risk factors for VTE. LMWH is also used as therapeutic anticoagulation for VTE during pregnancy and postpartum [9]. Several international organizations have proposed recommendations for thromboprophylaxis in pregnant women with symptomatic COVID-19, while puerperal women are not always included in these recommendations [3, 10]. In both Sweden and Norway, thromboprophylaxis guidelines for pregnant and puerperal women were implemented during the first months of the pandemic, and summarized in eTable 1 [11, 12]. Previous studies have shown that pregnant women infected with COVID-19 were at higher risk of severe disease [13, 14], adverse pregnancy outcomes, including severe maternal morbidity measures [15–18], as well as VTE during pregnancy or the postpartum. A study of over 129,000 deliveries in the United States in 2020, including more than 2,400 women with COVID-19, reported an increased VTE risk extending from pregnancy to six weeks postpartum [19]. Similarly, research from Scotland comparing pregnant individuals with and without COVID-19 found a significantly elevated risk of VTE among those infected [20]. Another U.S. cohort study examining women hospitalized for childbirth further reinforced this association, demonstrating a higher risk of VTE in those with COVID-19 [18]. However, these studies did not consider that some participants may have been prescribed thromboprophylaxis either due to symptomatic COVID-19 or other known risk factors, thus highly reducing their risk of VTE.

This large population-based registry study from Norway and Sweden aimed to investigate the association between COVID-19 and VTE in pregnant and puerperal women taking the use of anticoagulants into account.

## Materials and methods

### Study participants and setting

**Fig. 1:**
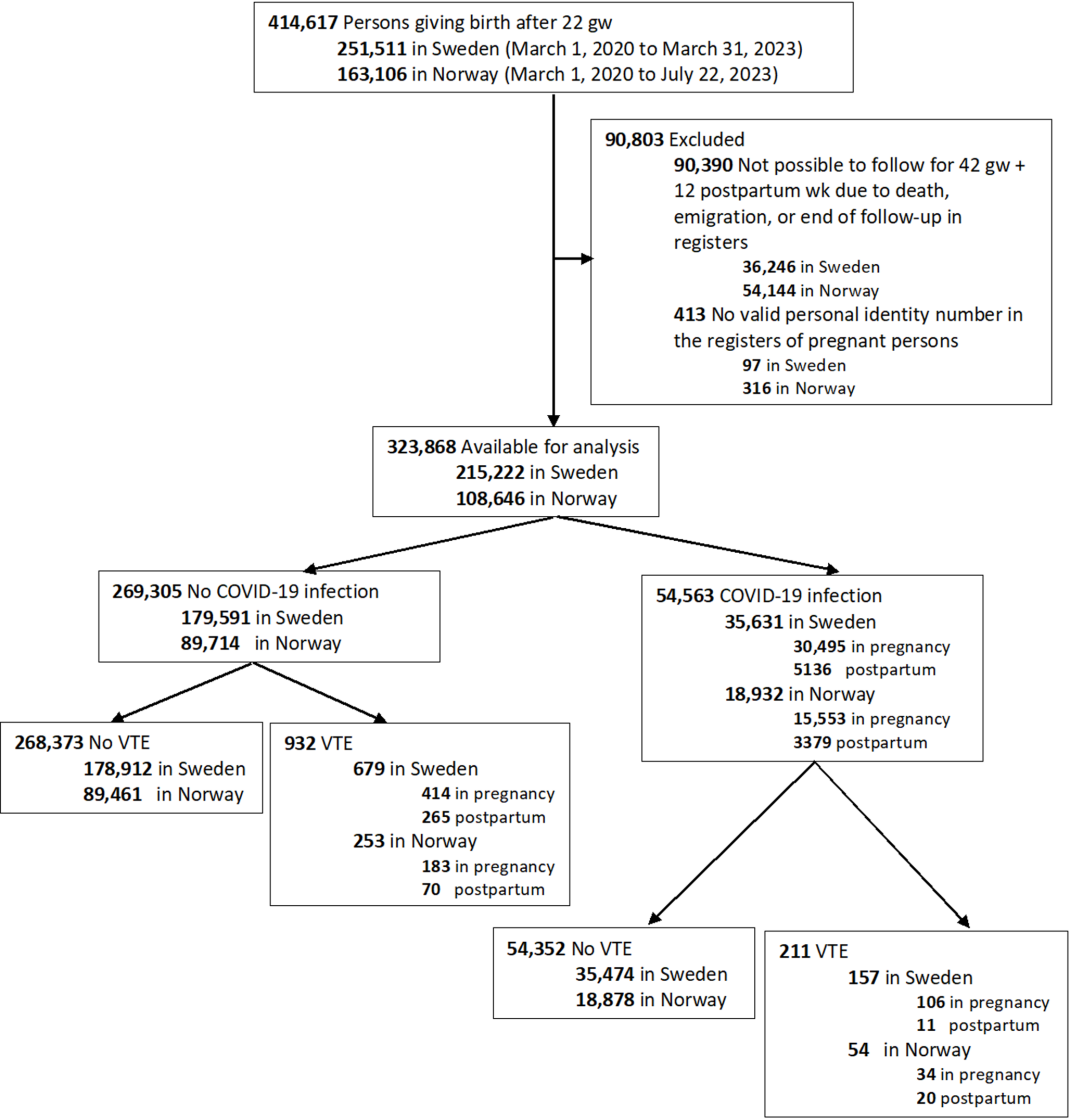
Selection of the study population in a study of COVID-19 and venous thromboembolism (VTE) in pregnancy and postpartum. *VTE* venous thromboembolism, *gw* gestational week, *wk* week

This was a population-based cohort study of all women giving birth at 22 or more gestational weeks between March 1, 2020, and March 31, 2023 (in Sweden) / July 22, 2023 (in Norway) identified in the Swedish Pregnancy Register [21] and Norwegian Birth Registry [22]. Exclusion criteria were: no valid personal identity number and lack of possibility to be followed in the national registers for 42 gestational weeks and 12 weeks postpartum (Fig. 1). Conception dates were estimated based on fetal ultrasonography or the last menstrual period. The first eligible date of conception was March 1, 2020, which was right before the start of the COVID-19 pandemic, and the last conception date with complete follow-up was March 18, 2022. The unique personal identity number was used to link national register data on exposure, outcome, and other covariates. The data sources are further described in the Online Supplementary Information.

### Exposures

Information on COVID-19 was identified from national infectious disease surveillance registers. COVID-19 infection during pregnancy and the postpartum period was defined as the first positive PCR test for SARS-CoV-2 between the date of conception and the date of delivery for the pregnancy period and between days 1–84 (12 weeks) after delivery for the postpartum period. The date of when the PCR test was performed, was used in the analysis as a proxy for onset of COVID-19 infection. Although limited by the absence of complete variant sequencing data, we identified four time periods when different variants of the SARS-CoV-2 virus were dominant: Index (1 March 2020–31 January 2021), Alpha variant (February 2021–June 2021), Delta variant (July 2021–December 2021), and Omicron variant (January 2022–March 2022) [23]. Testing strategies in Sweden and Norway [24, 25] are summarized in the Online Supplementary Information.

### Outcome

Based on previous validated register-based outcomes of VTE [26–28], VTE was defined as an in- or outpatient ICD-10 (International Classification of Diseases 10th revision) code of I26, I80, I81, I82, O223, O871, and O882 (Online resource eTable 2) in the pregnancy and national patient registers using the first record (exact date) identified during pregnancy or postpartum as the time of VTE. The specific codes for superficial vein thrombosis in pregnancy (O222) and postpartum (O870) were not included as our primary interest was in deep venous thromboembolism, which carries a higher risk of morbidity and mortality.

### Anticoagulants

Based on Anatomical Therapeutic Chemical classification codes (ATC: B01AA, B01AB, B01AF; Online Resource eTable 3), prophylactic and therapeutic anticoagulants (date of filled prescriptions) up to 3 years before, during, and 12 weeks after pregnancy, were identified from national prescription registers [29, 30].

### Covariates

Other characteristics were identified from the pregnancy and birth registers, Statistics Sweden/Norway [31, 32], and national vaccination registers [33] and included age at conception, early-pregnancy body mass index (BMI), early pregnancy smoking status (yes/no), region of birth (Scandinavia, Other European countries, Middle East/Africa, or other), education level (≤ 9 years, 10–12 years, and > 12 years), income (in tertiles), parity (nulliparous, multiparous), multiple pregnancy (yes/no), season of conception (March–April 2020, May–August 2020, September–December 2020, January–April 2021, May–August 2021, September-December 2021, January–March 2022), and COVID-19 vaccination status (at least one dose, yes/no).

### Statistical analyses

We used Cox regression analysis to evaluate hazard ratios of VTE according to COVID-19 infection in pregnancy or the postpartum period, separately. The time axis was gestational age in days for the analysis of VTE during pregnancy with the start of follow-up from the estimated date of conception. The time axis in the postpartum period analysis was days from delivery with the start of follow-up on day 1 after delivery. COVID-19 infection was entered as a time-varying exposure, and participants could contribute to both unexposed and exposed follow-up time. The analyses were censored at the date of VTE diagnosis, when a person had been prescribed an anticoagulant within 2 weeks from testing positive for COVID-19, end of study period (date of delivery for the pregnancy analyses/day 84 after delivery for the postpartum period analysis), whichever occurred first, adjusting for anticoagulants up to 3 years prior the current pregnancy, and including anticoagulant use during the follow-up as a time-varying exposure. The sandwich estimator of standard errors was used to account for clustering individuals with several pregnancies during the follow-up. We further adjusted for maternal age, BMI, smoking, region of birth, education, income, parity, multiple pregnancy, and season of conception. Since we lacked sufficient information to impute missing values for education, income, BMI, and smoking, we handled missing data using a missing indicator in the adjusted models. In a sensitivity analysis, individuals with a history of anticoagulants up to 3 years prior to pregnancy were excluded. In secondary analyses, to evaluate whether there was a particular increased risk of VTE shortly after testing positive for COVID-19, we estimated the risk of VTE in the first 2, 4, 8, 12, and 16 weeks following COVID-19 infection. In these analyses, participants were considered unexposed again after the end of the specific risk window. Further, the risk of VTE by different periods of infection (Index, alpha, Delta, or Omicron) was assessed. Lastly, to study whether COVID-19-infected women not vaccinated against COVID-19 were at different risk of VTE compared with women with at least one dose of vaccine before COVID-19 infection, we stratified the analyses on vaccination status at COVID-19 infection.

The data from each country were analyzed separately and subsequently meta-analyzed using a random-effects model, with heterogeneity estimated using the I^2^ statistic. Results are reported as unadjusted and adjusted hazard ratios with 95% confidence intervals (CI). Analyses were conducted using SAS version 9.4 (SAS Institute) and Stata version 18 (Statacorp, Texas).

## Results

### Exposure data

**Fig. 2:**
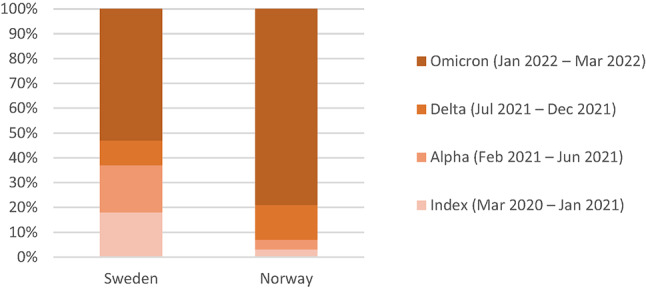
Proportions infected in different periods corresponding to dominating virus variants of the 54,563 COVID-19-infected women (35, 631 in Sweden and 18,932 in Norway)

In total, 323,868 women with a birth after 22 gestational weeks were included, and 46,048 (14.2%) of these had COVID-19 during pregnancy, while 8515 (2.6%) experienced COVID-19 during the 12 weeks postpartum. The median time from conception to infection in pregnancy was 145 days (IQR, 79–211) in Sweden and 138 days (IQR, 75–210) in Norway. The median time from the first postpartum day to infection among puerperal individuals was 47 days (IQR, 25–66) in Sweden and 48 days (IQR, 28–66) in Norway. While the highest proportion of COVID-19-infected women in both countries was seen during the Omicron period (Sweden: 53%; Norway: 79%), Sweden had a larger proportion of infected women during the Index (18%) and Alpha (19%) periods compared to Norway (3% in Index, and 4% in Alpha period) (Fig. 2).

### Maternal and pregnancy characteristics

Maternal and pregnancy characteristics were similar between COVID-19-infected and non-infected, and between Sweden and Norway, except for a higher proportion of missing data on smoking, education, and income in Norway, a higher proportion of women originating from the Middle East/Africa in Sweden, and a higher proportion of women with ≤ 9 years of education in Norway (Table 1).

**Table 1:** Maternal and pregnancy characteristics by COVID-19 infection status in pregnancy and 12 weeks postpartum in Sweden and Norway

	Sweden		Norway	
Characteristics	No COVID-19 infection *N* = 179,591	COVID-19 infection *N* = 35,631	No COVID-19 infection *N* = 89,714	COVID-19 infection *N* = 18,932
Maternal age at the estimated day of conception				
Mean (SD)—yr	30.9 (4.8)	31.0 (4.8)	30.8 (4.7)	31.0 (4.6)
Median (IQR)—yr	30.8 (27.7–34.0)	30.8 (27.8–34.1)	30.7 (27.6–33.8)	30.9 (27.8–34.1)
Range, min-max—yr	12.7–55.6	14.9–56.3	14.4–55.4	15.5–54.8
*Early-pregnancy BMI*				
Mean	25.5 (5.1)	25.8 (5.2)	25.0 (5.1)	25.0 (5.0)
Median (IQR)	24.5 (21.9–28.1)	24.6 (22.1–28.3)	23.8 (21.4–27.3)	23.9 (21.5–27.4)
Range, min-max	13.7–64.9	13.2–78.7	14.2–58.8	14.0-54.8
Missing—no. (%)	7 196 (4.0)	1 353 (3.8)	5 185 (5.8)	1 020 (5.4)
*Smoking in early pregnancy— no. (%)*				
Non-smoker	167,382 (93.2)	33,227 (93.3)	77,136 (86.0)	16,161 (85.4)
Smoker	5 596 (3.1)	1 014 (2.8)	1 527 (1.7)	274 (1.5)
Missing	6 613 (3.7)	1 390 (3.9)	11,051 (12.3)	2 497 (13.2)
*Region of birth—no. (%)*				
Scandinavia	127,301 (70.9)	25,829 (72.5)	67,437 (75.2)	13,218 (69.8)
Other European countries	13,742 (7.7)	2 956 (8.3)	9 578 (10.7)	2 479 (13.1)
Middle East/Africa	29,559 (16.5)	5 504 (15.4)	5 818 (6.5)	1 904 (10.1)
Other/missing	8 989 (5.0)	1 342 (3.8)	6 881 (7.7)	1 331 (7.0)
*Highest achieved education—no. (%)*				
≤9 years	10,891 (6.1)	1 689 (4.7)	11,723 (13.1)	2 792 (14.8)
10–12 years	55,679 (31.0)	11,538 (32.4)	16,694 (18.9)	3 846 (20.3)
>12 years	111,654 (62.2)	22,281 (62.5)	51,392 (57.3)	10,153 (53.6)
Missing	1 367 (0.8)	123 (0.3)	9 635 (10.7)	2 141 (11.3)
*Income —no. (%)*				
1st tertile	58,858 (32.8)	11,158 (31.3)	27,349 (30.5)	6 255 (33.0)
2nd tertile	57,323 (31.9)	12,693 (35.6)	27,993 (31.2)	6 073 (32.1)
3rd tertile	58,666 (32.7)	11,350 (31.9)	28,217 (31.5)	5 492 (29.0)
Missing	4 744 (2.6)	430 (1.2)	6 155 (6.9)	1 112 (5.9)
*Parity—no. (%)*				
Nulliparous	78,661 (43.8)	13,791 (38.7)	39,930 (44.5)	6 626 (35.0)
Multiparous	100,930 (56.2)	21,840 (61.3)	49,784 (55.5)	12,306 (65.0)
*Singletons—no. (%)*	177,145 (98.6)	35,232 (98.9)	88,425 (98.6)	18,711 (98.8)
*Covid-19 vaccination in pregnancy– no. (%)*				
Yes	63,786 (35.5)	13,376 (37.5)	23,926 (26.7)	6559 (34.7)
No	115,805 (64.5)	22,255 (62.5)	65,788 (73.3)	12,373 (65.4)

### Anticoagulants before, during, and after pregnancy

Table 2 displays the number and proportion of women with a filled prescription of an anticoagulant before, during, and up to 12 weeks postpartum. Overall, 3.2% had been prescribed an anticoagulant within 3 years before the current pregnancy, and 2.6% in Sweden and 1.5% in Norway had been prescribed an anticoagulant in pregnancy. Among COVID-19-infected pregnant women in Sweden, 7.6% had been prescribed an anticoagulant within two weeks from the onset of COVID-19 infection. The corresponding number in Norway was 0.3%. In puerperal women with COVID-19, 1.2% in Sweden and 0.03% in Norway had been prescribed an anticoagulant within 2 weeks from the onset of COVID-19.

**Table 2:** Prescriptions of anticoagulants according to COVID-19 infection before, during pregnancy and 12 weeks postpartum

Prescriptions of anticoagulants†	Sweden		Norway	
	No COVID-19 infection *N* = 179,591	COVID-19 infection *N* = 35,631	No COVID-19 infection *N* = 89,714	COVID-19 infection *N* = 18,932
*At least one prescription– no. (%)*				
Within 3 years before pregnancy	5 661 (3.2)	1 207 (3.4)	2830 (3.2)	709 (3.7)
During pregnancy only	2 664 (1.5)	3 035 (8.5)	1270 (1.4)	322 (1.7)
During postpartum only	7 416 (4.1)	1 564 (4.4)	1822 (2.0)	104 (0.5)
During pregnancy *and* postpartum	1 394 (0.8)	567 (1.6)	412 (0.5)	27 (0.1)
During pregnancy *or* postpartum	11,474 (6.4)	5 166 (14.5)	3504 (3.9)	453 (2.4)
*Prescription within 2 weeks of onset of COVID-19 infection– no. (%)*				
During pregnancy	NA	2 718 (7.6)	NA	54 (0.3)
Postpartum	NA	430 (1.2)	NA	< 5

†Includes ATC codes B01AB01, B01AB04, B01AB05, B01AB09, B01AB10 (during pregnancy) and B01AB01, B01AB04, B01AB05, B01AB09, B01AB10, B01AA03, B01AF01, B01AF02, B01AF03 (prior pregnancy and postpartum)

### VTE in pregnancy

**Fig. 3:**
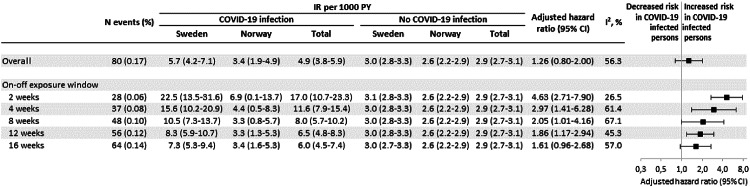
The association between COVID-19 infection and VTE in pregnancy. The total number of VTE events during pregnancy in both countries, the incidence rate per 1000 person-years (PY) by country and total, and adjusted hazard ratio with 95% confidence intervals (CI), in a meta-analysis of both countries. Censored at date of VTE, time of prescription of anticoagulants within 2 weeks of onset from COVID-19 infection, or end of study period, whichever occured first, and adjusted for maternal age at the estimated day of conception, region of birth, highest achieved education, income, parity, multiple pregnancies, calendar time of conception, early-pregnancy BMI, smoking in early pregnancy, anticoagulants up to 3 years of current pregnancy, and anticoagulants as time-varying covariate during follow-up

Among COVID-19-infected pregnant women, 80 (0.17%) had a VTE after being infected. The median time from infection in pregnancy to VTE was 22 days (IQR, 6–70) in Sweden and 92 days (IQR, 27–172) in Norway. Among all VTE registrations, 87% were registered as out-patient diagnoses and 13% were in-patient diagnoses. In Sweden, COVID-19 during pregnancy was associated with an increased risk of VTE (incidence rate, 5.7 vs. 3.0 per 1000 person-years; aHR 1.51 (95% CI 1.14–2.02). In Norway, the incidence rate of VTE after infection was 3.4 vs. 2.6 per 1000 person-years; aHR 0.93 (95% CI 0.53–1.62). The incidence rate for both countries combined was 4.9 among infected vs. 2.9 among non-infected, per 1000 person-years, and in the meta-analysis, the aHR was 1.26 (95% CI 0.80-2.00), I^2^ = 56.3% (Fig. 3, eFigure 1 [unadjusted HR, country-specific estimates] and eFigure 2 [adjusted HR, country-specific estimates]). Exclusion of individuals with a history of anticoagulants prior pregnancy, yielded similar estimates with aHR 1.60 (95% CI 1.18–2.16) in Sweden and aHR 1.00 (95% CI 0.56–1.80) in Norway. The aHR for both countries combined was 1.36 (95% CI 0.87–2.11), I^2^ = 49.1%.

In meta-analyses of 2, 4, 8, 12, and 16-week exposure windows during pregnancy, the highest hazard was seen during the first 2 weeks following infection, with an aHR of 4.63 (95% CI 2.71–7.90), I^2^ = 26.5%, and the risk remained elevated until 12 weeks following infection: aHR 1.86 (95% CI 1.17–2.94), I^2^ = 45.3% (Fig. 3, eFigure 1 and eFigure 2).

The highest proportion of VTE among COVID-19-infected pregnant individuals in Sweden was seen during the Alpha period (*n* = 20, 0.37%), corresponding to an aHR of 5.05 (95% CI 2.80–9.12). Similar proportions of VTE among COVID-19 infected women were seen for the Index (0.13%), Delta (0.11%), and Omicron (0.14%) periods with aHR estimates ranging between 1.18 and 2.02 with 95% CIs overlapping 1. In Norway, the proportion of VTE among COVID-19-infected pregnant women was similar for the Alpha (0.17%), Delta (0.14%), and Omicron (0.13%) periods, while no VTE was diagnosed during the Index period. There were too few VTE events for each variant period to estimate hazards for each period (data not tabulated).

In analyses stratified by vaccination status at COVID-19 infection, an aHR of 1.60 (95% CI 1.18–2.16) was seen among non-vaccinated compared to an aHR of 0.95 (95% CI 0.46–1.95) among vaccinated in Sweden. In Norway, the corresponding numbers were 0.28 (95% CI 0.04–1.99) among non-vaccinated and 1.11 (95% CI 0.62–1.98) among vaccinated.

### VTE postpartum

In both countries, 6 (0.07%) puerperal women were diagnosed with VTE after COVID-19 infection. The median time from infection to VTE was 13 days (IQR, 8–24) in Sweden and 22 days (IQR, 14–30) in Norway. In a meta-analysis of both countries, COVID-19 postpartum was associated with an increased hazard of VTE with an aHR of 5.17 (95% CI 2.50-10.69), I^2^ = 0.0% (data not tabulated). Country-specific results are not shown due to national privacy regulations for presenting small numbers.

## Discussion

In this large population-based registry study, we found that the risk of VTE increased following COVID-19 infection during pregnancy, peaking in the first two weeks and remaining elevated for up to 3 months. We also observed an association between COVID-19 and postpartum VTE, although these findings should be interpreted cautiously due to small sample sizes.

Our results align with other studies on pregnant [18–20] and postpartum women [19]. Bruno et al. studied 129,604 deliveries in the United States in 2020, including 2432 women with COVID-19 [19]. They found an increased VTE risk from pregnancy to six weeks postpartum (aHR 2.62, 95% CI 1.60–4.29). Similarly, a Scottish study comparing 4072 pregnant women with COVID-19 to 12,216 non-infected women reported an adjusted odds ratio (aOR) of 2.53 (95% CI 1.47–4.35) for VTE [20]. Another U.S. cohort of 6380 women with COVID-19 hospitalized for childbirth showed an aOR of 3.43 (95% CI 2.01–5.82) [18]. The novelty of this study is the population-based design including all test-positive COVID-19-infected pregnant and puerperal women, the possibility to take the timing of infection into account, as well as the inclusion of anticoagulants in the analysis which has not been done previously. This is of importance as pregnant and puerperal women are a high-risk group for thrombotic events and are often prescribed prophylactic anticoagulants due to other reasons than COVID-19 infection which may influence estimates of VTE risk after COVID-19 infection. The baseline risk of VTE in this study was higher than previously described in the Scandinavian countries [7, 8, 26]. This discrepancy may stem from several factors, including differences in study populations, where for instance, pro-thrombotic factors such as increasing maternal age and BMI have steadily risen during the last decade, case ascertainment methods, and registry-based identification of events.

While the overall hazard ratio for VTE following COVID-19 infection was modest, this estimate reflects an average over the entire follow-up period and may obscure short-term elevations in risk. This study focused on different time windows after infection and found the highest VTE risk within the first two weeks. A study of 48 million non-pregnant adults in England and Wales also found that VTE risk was highest during the first week after diagnosis (aHR 33.2, 95% CI 31.3–35.2) and remained elevated (aHR 1.80, 95% CI 1.50–2.17) up to one year later [34]. A case series study found that excluding VTE cases recorded on the first day of COVID-19 diagnosis reduced the early relative incidence but showed elevated risk up to 28 days [35]. These findings suggest that high HRs observed in the first days of COVID-19 infection may be inflated by reverse causality attributable to the ascertainment of COVID-19 at the time of diagnosis for VTE. However, reverse causation does not explain the persistent risk up to three months post-infection, which instead could be due to a prolonged post-infection inflammatory response. The mechanisms behind this response, whether endothelial, leukocyte, or other inflammation components, remain unclear [36] and require further clinical investigation.

Although we found an increased VTE risk in both pregnancy and postpartum periods after COVID-19 infection, the absolute number of cases was low in Sweden and Norway, possibly due to few severe COVID-19 cases among pregnant women in both countries [37]. Swedish estimates were consistently higher than Norwegian estimates, but both countries followed a pattern of highest risk during the first weeks of infection, followed by a gradual decrease over time. Variations in testing strategies, the intensity of the pandemic, anticoagulant prescriptions, and adherence to thromboprophylaxis guidelines may explain differences between the countries. In our study, fewer women in Norway were infected during the Index and Alpha periods compared to Sweden, whereas more Norwegian women were infected during the Omicron period. A study on maternal outcomes associated with Omicron and previous variants found that Omicron-infected women were less likely to develop VTE, although anticoagulant use was not considered [38]. These differences could contribute to the varying estimates between the countries. Vaccination status may also explain the differences. Sweden had a higher proportion of unvaccinated women during infection compared to Norway, which may have influenced VTE risk. To account for this heterogeneity, we used a random-effects meta-analysis.

Previous studies (100–1220 participants) have shown that VTE risk is linked to disease severity [14, 39, 40]. Unfortunately, our registry data lacked information on COVID-19 symptoms and clinical severity, so we could not investigate whether disease severity or sequenced virus variants contributed to the differences between Sweden and Norway. Future studies with large samples of pregnant women, symptom reports, and virus variant sequencing should explore these factors.

### Strengths and limitations

Our study had several strengths. We included information on anticoagulant use, allowing us to identify women at high risk of new thrombotic events. Anticoagulants may have been prescribed for pre-existing VTE risk factors or due to COVID-19, reducing the risk of VTE acquisition. Additionally, linking national registers from two countries, rather than relying on insurance claims databases [19] or hospitalized cohorts [18], provided a more comprehensive dataset. The mandatory reporting of COVID-19 and vaccination status in these registers also enhanced the generalizability of our findings.

The findings should be interpreted in light of several limitations. First, as this is an observational study, we cannot rule out the possibility of unknown or residual confounding, or effect modification by other conditions, such as coagulation disorders, for which data were not available. Future research incorporating comorbidities and mode of delivery could provide further clarity on the interplay between COVID-19 and VTE risk in pregnancy and the postpartum period. Second, despite including data from nearly all pregnant and postpartum women during the pandemic’s first three years, VTE was rare, limiting our statistical power to examine risk by time postpartum and differences by virus variants. Nonetheless, our study’s sample size exceeded that of previous studies [18–20]. Third, while our study included anticoagulants, only prescribed ones were recorded. This could result in misclassification, as women treated with anticoagulants in hospitals without outpatient prescriptions would not be categorized as users, potentially underestimating VTE risk. Further, while the thromboprophylaxis guidelines in Sweden and Norway are in line with several other international recommendations [3], we acknowledge that variability in anticoagulation practices across different countries may reduce the generalizability of our findings to other healthcare settings. Lastly, although we were unable to validate our VTE outcome definition using medical records, two previous studies validated a similar definition and reported high accuracy [27, 28]. However, misclassification in register data cannot be excluded, despite the data sources being considered of high validity [21, 26, 29, 41].

## Conclusions

COVID-19 during pregnancy or the postpartum period was associated with a higher risk of VTE, even when accounting for anticoagulants. While the absolute VTE risk was low, our findings should contribute to the individual risk assessment when evaluating the need for prophylactic anticoagulants during infection in pregnancy and postpartum.

## Electronic supplementary material

Below is the link to the electronic supplementary material.

Supplementary Material 1
