# Supplementary material for: COVID-19 infection increases the risk of venous thromboembolism during pregnancy and the postpartum period
Source: Eur J Epidemiol. 2025 Jul 11;40(7):779–88. doi: 10.1007/s10654-025-01268-z (PMC12304068; doi:10.1007/s10654-025-01268-z)
Supplement: Supplementary file 1 — Supplementary Material 1 [file 10654_2025_1268_MOESM1_ESM.docx]

**Supplementary Information**

**Supplement to:** COVID-19 infection increases the risk of venous thromboembolism during pregnancy and the postpartum period, **European Journal of Epidemiology**

**Authors:** Anne K. Örtqvist, MD, PhD, Jonas Söderling, PhD, Maria C. Magnus, PhD, Stine Kjaer Urhoj, PhD, Siri E. Håberg, MD, PhD, Olof Stephansson, MD, PhD

**Corresponding author:**

Anne K. Örtqvist, MD, PhD

Email: [Anne.ortqvist@ki.se](mailto:Anne.ortqvist@ki.se)

Postal address: Clinical Epidemiology Division, Department of Medicine

Karolinska Universitetssjukhuset, Solna, T2:02, SE-171 76 Stockholm, Sweden

Telephone: +46 737 355 309

| **Table of contents** | |  |
| --- | --- | --- |
| Data sources and linkages in Sweden | | *p. 2* |
| Data sources and linkages in Norway | | *p. 3* |
| Testing strategies | | *p. 4* |
| **eTable 1** | National guidelines of thromboprophylaxis due to COVID-19 | *p. 5* |
| **eTable 2** | Diagnostic codes for venous thromboembolism | *p. 6* |
| **eTable 3** | ATC-codes of anticoagulants | *p. 7* |
| **eFigure 1** | The unadjusted hazard ratio for COVID-19 infection and the risk of VTE during pregnancy in Sweden, Norway, and combined | *p. 8* |
| **eFigure 2** | The adjusted hazard ratio for COVID-19 infection and the risk of VTE during pregnancy in Sweden, Norway, and combined | *p. 9* |
| **References** |  | *p. 10* |
|  |  |  |

**Data sources and linkages in Sweden**

*The Swedish Pregnancy Register (SPR)*

Data in this study was provided through the Swedish Pregnancy Register (SPR) [1]. This quality register was initiated in 2013 and includes 99% of all births in Sweden (20 of 21 regions). Demographical, reproductive, and maternal health care data, starting at the first visit to the antenatal care clinic around the ninth gestational week, are transferred from electronic medical records within 24 hours from a reported birth. The register includes information on birth outcomes, maternal background characteristics, health during pregnancy, and maternal and neonatal outcomes.

*Registers at the National Board of Health and Welfare*

Information on anticoagulants was collected based on Anatomical Therapeutic Chemical (ATC) codes from the Prescribed Drug Register [2] with a lookback of 3 years from March 2020 (**eTable 2**). The outcome of venous thromboembolism was collected from both the SPR and the National Patient Register [3] according to the International Classification of Diseases tenth revision (ICD-10) diagnosis codes **(eTable 3).**

*Registers at the Public Health Agency of Sweden*

From the start of the pandemic in March 2020, the SPR has been linked to the Swedish Register for Communicable Diseases (SmiNet) and the national vaccination register to retrieve regularly updated data on PCR-verified SARS-CoV-2 tests and vaccination coverage among all birthing women in the SPR.

SmiNet is the national register for communicable diseases. On February 1, 2020, SARS-CoV-2 was included in the Swedish Communicable Diseases Act, making it mandatory to report all laboratory-confirmed Polymerase Chain Reaction (PCR) cases within 24 hours to the register. From this register, we used information on positive tests as well as the date of a positive PCR test. Information on key elements regarding the Swedish pandemic strategy has been discussed in detail in papers by Ludvigsson JF [4,5].

As of January 1, 2013, healthcare providers must report all vaccinations administered within the Swedish vaccination programs to the Swedish vaccination register, held by the Public Health Agency of Sweden. The register includes the type and date of all COVID-19 vaccinations. Up to the end of May 2021 in Sweden, vaccination was only recommended to pregnant women with a high risk of severe COVID-19. Thereafter, a general recommendation for pregnant women to get vaccinated was issued, although women were recommended to wait for vaccinations until after 12 weeks of gestation. As the availability of vaccines was restricted initially, vaccination was still being prioritized based on age (oldest first). From August 2021, however, the vaccine was available to all above 18 years of age. More information on COVID-19 vaccinations among pregnant and birthing women in Sweden and Norway can be found in recent publications [6-8].

*Registers at Statistics Sweden*

Education, income (average individual income from work during 2017-2019 in tertiles), and country of birth were collected from the Total Population Register, Education Register and Income and tax register at Statistics Sweden.

**Data sources and linkages in Norway**

*The* *Emergency Preparedness Register for Covid-19*

Data in this study were provided through the Emergency Preparedness Register for Covid-19 (Beredt C19) administered by the Norwegian Institute of Public Health, according to the Health Preparedness Act §2-4 [9].This registry was established in 2020 to provide authorities with up-to-date information on the prevalence, causal relationships, and consequences of the COVID-19 epidemic in Norway. Beredt C19 includes information already collected in the healthcare service, national health registries, and administrative registers with information about the Norwegian population. The data subjects' right is safeguarded as they can contact the data controller for the different sources included in Beredt C19 in the usual way. Through Beredt C19 we used data from the following sources:

*The Medical Birth Registry of Norway (MBRN)*

The Norwegian Medical Birth Registry includes information on all pregnancies ending in gestational week 12 or later [10]. The registry includes information on birth outcomes in addition to maternal background characteristics, health during pregnancy, pregnancy outcomes, and neonatal health.

*National Patient Register*

The Norwegian patient registry includes individual-level information on all contacts with specialist health-care services. Information registered includes admission and discharge dates, and diagnostic codes during the hospital stay, and for outpatient visits. The outcome of venous thromboembolism was according to the International Classification of Diseases tenth revision (ICD-10) diagnosis codes **(eTable 2).**

*Norwegian Surveillance System for Communicable Diseases (MSIS)*

There is mandatory reporting of selected infectious diseases to this surveillance register [11]. Reporting of all COVID-19 tests is mandatory, and this register contains the date of testing and test results.

*The Norwegian Immunisation Register (SYSVAK)*

SYSVAK is a register of vaccines in the Norwegian vaccination program, with mandatory registrations of all COVID-19 vaccinations (dates and type). Up to the middle of August 2021 in Norway, vaccination was only recommended to pregnant women with a high risk of severe COVID-19. Thereafter, a general recommendation for pregnant women to get vaccinated was issued, although women were recommended to wait for vaccinations until after 12 weeks of gestation. Since January 2022, women in Norway have been recommended vaccination regardless of trimester.

*Statistics Norway (SSB)*

Administrative data is mandatorily reported to Statistics Norway [12]. We used information from this database on household income in 2018, type of education, and years of education completed by 2019.

*Norwegian Prescription Database (NorPD)*

All dispensed prescriptions from pharmacies from January 1^st,^ 2004 are included in the Norwegian Prescription Database. All medications are coded according to the Anatomical Therapeutic Chemical system. From this registry, we obtained information on all anticoagulants with a lookback of 3 years from March 2020 (**eTable 3**).

**Testing strategies**

In Sweden, SARS-CoV-2 was included in the Swedish Communicable Disease Act on 1^st^ February 2020, making all laboratory-confirmed polymerase chain reaction (PCR) cases of SARS-CoV-2 mandatory to report within 24 hours to SmiNet at the Public Health Agency of Sweden. Negative test results are unfortunately not available on a national level. A non-universal population testing strategy was implemented including outpatient testing and contact tracing, starting in June 2020 and ongoing to January 2022. This type of testing mostly included symptomatic individuals, but it could also include individuals tested before and after travels, or after contact with other test-positive individuals and individuals subject to workplace testing (e.g. healthcare workers). In 23 of the 39 delivery hospitals covered by the Swedish Pregnancy Register, universal testing of all women admitted for labor or pregnancy in-patient care, independent of their current and previous medical history and COVID-19 symptoms or not, was performed.

In Norway, mandatory registration of all PCR tests for SARS-CoV-2 was implemented in the Norwegian Surveillance System for Communicable Diseases on 31^st^ January 2020. As Norway has not had universal testing of pregnant or delivering women, a non-universal testing strategy was operating throughout the study period. Testing was predominantly conducted based on symptoms to confirm or exclude SARS-CoV-2 infection, with some additional testing conducted for particular reasons such as contact with infected persons, or mandatory testing due to travel or work. Information was available on all conducted tests, regardless of positive or negative results.

From mid-January 2022, the testing has decreased due to new recommendations. Individuals are no longer required to get tested, but to stay at home if one has symptoms. However, healthcare workers, and pregnant women with symptoms of COVID-19 in contact with healthcare, are still recommended to get tested, and several delivery hospitals still test pregnant women upon admission to the delivery ward independent of symptoms.

Concerning the varying testing strategies in the two countries over time, the final models were adjusted for the calendar time of conception.

**eTable 1.** National guidelines of thromboprophylaxis due to COVID-19 in pregnancy and postpartum^a^

|  | **Sweden** [13] | **Norway** [14] |
| --- | --- | --- |
| **Outpatients with COVID-19** | | |
| Pregnancy | *Individualized risk assessment:* 1) asymptomatic or mild disease with ≤2 risk points^b^ for VTE then NO thromboprophylaxis necessary  2) asymptomatic or mild disease with ≥2 risk points^b^ for VTE, and no contradictions to treatment, recommend thromboprophylaxis until full mobilization and at least 2 weeks of absence from acute symptoms. | *Individualized risk assessment in women with* asymptomatic or mild disease without the need for hospitalization, should be considered for thromboprophylaxis by usual criteria and advice on physical activities, to avoid dehydration and support stockings in the third trimester. |
| Postpartum |  | Women who test positive at delivery should be considered for thromboprophylaxis for a minimum of 10 days after delivery, especially if there are active COVID-19 symptoms or other risk factors. |
| **Hospitalized due to COVID-19** | | |
| Pregnancy | Patients with moderate to severe disease, and immobilized patients are recommended thromboprophylaxis during the hospital stay and another 2 weeks and until full mobilization. | Patients with moderate to severe disease who are hospitalized are recommended thromboprophylaxis until full mobilization.  Patients with serious COVID-19 can be considered for higher doses until 14 days after discharge or 6 weeks postpartum. |
| Postpartum |  |  |
| **Hospitalized due to other reason than COVID-19, but tested positive for SARS-CoV-2** | | |
| Pregnancy | Individualized risk assessment, consider thromboprophylaxis if immobilized and/or another risk factor for VTE | Individualized risk assessment, consider thromboprophylaxis if immobilized and/or another risk factor for VTE. |
| Postpartum |  |  |

**^a^** Up to 6 weeks postpartum

^b^ Risk points for venous thromboembolism add to a risk score, where ≥2 points lead to thromboprophylaxis with different duration (7 days till approximately 6 weeks), time of initiation (early antepartum, temporary during pregnancy, or postpartum), and dose (normal vs high) depending on the risk score.

*1 point:* heterozygous FV Leiden mutation, heterozygous prothrombin mutation, age>40 years, pre-pregnancy BMI ≥30 and <40, VTE in 1^st^ relative <50 years of age, inflammatory bowel disease, hyperhomocysteinemia, and postpartum preeclampsia, placental abruption, caesarean section, blood transfusion, intrauterine foetal death, severe infection

*2 points*: protein S-deficiency, protein C-deficiency, immobilization, pre-pregnancy BMI >40

*3 points:* homozygous FV Leiden mutation, homozygous prothrombin mutation, double mutation

*4 points:* previous VTE, antiphospholipid syndrome (APS) without VTE (OAPS), ovarian hyperstimulation syndrome.

Individuals considered very high risk independent of other risk factors: continuous prophylactic anticoagulation, antithrombin-deficiency, repeated thrombotic events, APS with VTE (TAPS), sequelae from previous VTE, COVID-19 with oxygen treatment are recommended high dose thromboprophylaxis

***eTable 2.*** 10^th^ revision of the International Classification of Disease (ICD-10) of VTE, as suggested in a study by Abdul Sultan et al [15].

| **ICD 10** | **Diagnosis** | **Sweden**  **n (%)** | **Norway**  **n (%)** |
| --- | --- | --- | --- |
| I260 | Pulmonary embolism with mention of acute cor pulmonale | 4 (1) | 3 (1) |
| I269 | Pulmonary embolism without mention of acute cor pulmonale | 140 (18) | 52 (18) |
| I801 | Phlebitis and thrombophlebitis of femoral vein | 11 (1) | 9 (3) |
| I802 | Phlebitis and thrombophlebitis of other deep vessels of lower extremities | 68 (9) | 59 (20) |
| I803 | Phlebitis and thrombophlebitis of lower extremities, unspecific | 91 (11) | 9 (3) |
| I808 | Phlebitis and thrombophlebitis of other sites | 61 (8) | 14 (5) |
| I809 | Phlebitis and thrombophlebitis of unspecified site | 31 (4) | 20 (7) |
| I81 | Portal vein thrombosis | 10 (1) | 1 (0) |
| I820 | Budd-Chiari syndrome | 3 (0) | 1 (0) |
| I821 | Thrombophlebitis migrans | 1 (0) | 0 (0) |
| I822 | Embolism and thrombosis of vena cava | 2 (0) | 2 (0) |
| I823 | Embolism and thrombosis of renal vein | 1 (0) | 1 (0) |
| I828 | Embolism and thrombosis of other specified veins | 19 (2) | 22 (8) |
| I829 | Embolism and thrombosis of unspecified vein | 16 (2) | 30 (10) |
| O223 | Deep phlebothrombosis in pregnancy | 240 (30) | 40 (14) |
| O871 | Deep phlebothrombosis in the puerperium | 39 (5) | 14 (5) |
| O882 | Obstetric blood-clot embolism | 59 (7) | 11 (4) |
| **Total** |  | 796 | 288 |

***eTable 3.*** Anatomical Therapeutic Chemical classification (ATC) codes of prophylactic and therapeutic anticoagulants before, during, and after pregnancy

| **ATC codes** | **Description** |  |
| --- | --- | --- |
| **B01AA** | **Vitamin K antagonist** |  |
| B01AA03 | Warfarin (Waran) | Before and after pregnancy |
| **B01AB** | **Heparin group*** |  |
| B01AB01 | Heparin | Before, during and after pregnancy |
| B01AB04 | Dalteparin (Fragmin) | Before, during and after pregnancy |
| B01AB05 | Enoxaparin (Klexane) | Before, during and after pregnancy |
| B01AB09 | Danaparoid (Orgaran) | Before, during and after pregnancy |
| B01AB10 | Tinzaparin (Innohep) | Before, during and after pregnancy |
| **B01AF** | **Factor Xa inhibitors** |  |
| B01AF01 | Rivaroxaban (Xarelto) | Before and after pregnancy |
| B01AF02 | Apixaban (Eliquis) | Before and after pregnancy |
| B01AF03 | Edoxaban (Lixiana) | Before and after pregnancy |

*B01AB02: Antithrombin III should not be included as thromboprophylaxis treatment solely, as it is given during labor to women with hereditary antithrombin deficiency.

**eFigure 1.** The unadjusted hazard ratio for COVID-19 infection and the risk of VTE during pregnancy in Sweden, Norway, and combined

Censored at time of prescription of anticoagulants within 2 weeks of onset from COVID-19 infection and adjusted for anticoagulants within 3 years of pregnancy.

**eFigure 2.** The adjusted hazard ratio for COVID-19 infection and the risk of VTE during pregnancy in Sweden, Norway, and combined

Censored at time of prescription of anticoagulants within 2 weeks of onset from COVID-19 infection and adjusted for anticoagulants within 3 years of pregnancy, maternal age at the estimated day of conception, early-pregnancy BMI, smoking in early pregnancy, region of birth, highest achieved education, income, parity, multiple pregnancies, calendar time of conception, and anticoagulants as a time-varying covariate during follow-up.

**References**

1. Stephansson O, Petersson K, Bjork C, Conner P, Wikstrom AK. The Swedish Pregnancy Register - for quality of care improvement and research. Acta obstetricia et gynecologica Scandinavica. 2018;97(4):466-476.

2. Wallerstedt SM, Wettermark B, Hoffmann M. The First Decade with the Swedish Prescribed Drug Register - A Systematic Review of the Output in the Scientific Literature. Basic & clinical pharmacology & toxicology. 2016;119(5):464-469.

3. Ludvigsson JF, Andersson E, Ekbom A, et al. External review and validation of the Swedish national inpatient register. BMC Public Health. 2011;11:450.

4. Ludvigsson JF. The first eight months of Sweden's COVID-19 strategy and the key actions and actors that were involved. Acta paediatrica (Oslo, Norway : 1992). 2020;109(12):2459-2471.

5. Ludvigsson JF. How Sweden approached the COVID-19 pandemic: Summary and commentary on the National Commission Inquiry. Acta paediatrica (Oslo, Norway : 1992). 2023;112(1):19-33.

6. Ortqvist AK, Dahlqwist E, Magnus MC, et al. COVID-19 vaccination in pregnant women in Sweden and Norway. Vaccine. 2022;40(33):4686-4692.

7. Magnus MC, Ortqvist AK, Dahlqwist E, et al. Association of SARS-CoV-2 Vaccination During Pregnancy With Pregnancy Outcomes. Jama. 2022;327(15):1469-1477.

8. Norman M, Magnus MC, Soderling J, et al. Neonatal Outcomes After COVID-19 Vaccination in Pregnancy. Jama. 2024;331(5):396-407.

9. Norwegian Institute of Public Health. Emergency preparedness register for COVID-19 [homepage on the Internet] 2020; [cited 3 Jul 2024] Available from: <https://www.fhi.no/en/id/corona/coronavirus/emergency-preparedness-register-for-covid-19/>

10. Norwegian Institute of Public Health. Medical Birth Registry of Norway [homepage on the Internet]; [cited 3 Jul 2024] Available from: <https://www.fhi.no/en/ch/medical-birth-registry-of-norway/>

11. Norwegian Institute of Public Health. Norwegian Surveillance System for Communicable Diseases [homepage on the Internet]; [cited 3 Jul 2024] Available from: <https://www.fhi.no/en/ou/msis/>

12. Statistics Norway [homepage on the Internet]; [cited 3 Jul 2024] Available from: <https://www.ssb.no/en/>

13. Swedish Society of Obstetrics and Gynecology (SFOG). Guidelines regarding thromboprophylaxis in pregnancy due to COVID-19 [homepage on the Internet, webpage in Swedish]; [cited 3 Jul 2024] Available from: <https://www.sfog.se/media/337859/hemostasutredning-och-behandling-av-venoes-tromboembolism-vte-samt-trombosprofylax-vid-covid-19-hos-obstetriska-patienter-final.pdf>

14. Norsk gynekologisk forening. Coronavirus during pregnancy and postpartum. [homepage on the Internet, webpage in Norwegian]; [cited 3 Jul 2024] Available from: [https://www.legeforeningen.no/foreningsledd/fagmed/norsk-gynekologisk-forening/veiledere/veileder-i-fodselshjelp/koronavirus-ved-svangerskap-og-fodsel/.](https://www.legeforeningen.no/foreningsledd/fagmed/norsk-gynekologisk-forening/veiledere/veileder-i-fodselshjelp/koronavirus-ved-svangerskap-og-fodsel/)

15. Abdul Sultan A, West J, Stephansson O, et al. Defining venous thromboembolism and measuring its incidence using Swedish health registries: a nationwide pregnancy cohort study. BMJ open. 2015;5(11):e008864.
